# MicroRNA and mRNA interactions coordinate the immune response in non-lethal heat stressed *Litopenaeus vannamei* against AHPND-causing *Vibrio parahaemolyticus*

**DOI:** 10.1038/s41598-019-57409-4

**Published:** 2020-01-21

**Authors:** Pakpoom Boonchuen, Benedict A. Maralit, Phattarunda Jaree, Anchalee Tassanakajon, Kunlaya Somboonwiwat

**Affiliations:** 1 Center of Excellence for Molecular Biology and Genomics of Shrimp, Department of Biochemistry, Faculty of Science, Chulalongkorn University, Bangkok, Thailand; 2 Philippine Genome Center, University of the Philippines, Diliman, Quezon City, Philippines; 3 National Institute of Molecular Biology and Biotechnology, College of Science, University of the Philippines, Diliman, Quezon City, Philippines; 4 Institute of Molecular Biosciences, Mahidol University, Salaya, Nakhon Pathom, Thailand; 5 Omics Science and Bioinformatics Center, Faculty of Science, Chulalongkorn University, Bangkok, Thailand

**Keywords:** Biochemistry, Immunology, Molecular biology

## Abstract

While *Vibrio parahaemolyticus* (VP_AHPND_) has been identified as the cause of early mortality syndrome (EMS) or acute hepatopancreatic necrosis disease (AHPND) in shrimp, mechanisms of host response remain unknown. Understanding these processes is important to improve farming practices because this understanding will help to develop methods to enhance shrimp immunity. Pre-treatment of shrimp with 5-minute chronic non-lethal heat stress (NLHS) for 7 days was found to significantly increase *Litopenaeus vannamei* survival against VP_AHPND_ infection. To elucidate the mechanism involved, mRNA and miRNA expression profiles from the hemocyte of *L. vannamei* challenged with VP_AHPND_ after NLHS with corresponding control conditions were determined by RNA-Seq. A total of 2,664 mRNAs and 41 miRNAs were differentially expressed after the NLHS treatment and VP_AHPND_ challenge. A miRNA-mRNA regulatory network of differentially expressed miRNAs (DEMs) and differentially expressed genes (DEGs) was subsequently constructed and the interactions of DEMs in regulating the NLHS-induced immune-related pathways were identified. Transcriptomic data revealed that miRNA and mRNA interactions contribute to the modulation of NLHS-induced immune responses, such as the prophenoloxidase-activating system, hemocyte homeostasis, and antimicrobial peptide production, and these responses enhance VP_AHPND_ resistance in *L. vannamei*.

## Introduction

During its first outbreak, the devastating effect of early mortality syndrome (EMS) on the global shrimp industry was primarily caused by the lack of information regarding the disease and its causative agent^1^. Later studies that were focused on mitigating this disease eventually identified the cause to be a toxin-harboring *Vibrio parahaemolyticus*, which causes Acute hepatopancreatic necrosis disease (AHPND) (VP_AHPND_)^2^. This highlights how understanding disease etiology and a subsequent elucidation of host response can help to mitigate the effects of an outbreak. Understanding the functions of the shrimp immune system during disease progression is thus expected to create opportunities for the development of effective and efficient management strategies for VP_AHPND_ infection. Likewise, information regarding the molecular mechanisms of AHPND tolerance can lead to platforms for the development of AHPND-resistant shrimp through selective breeding using markers mined from transcriptome analyses at different rearing conditions.

It has been established that heat shock proteins (Hsps) and other immune-related genes in shrimp are up-regulated after infection with Vibrio spp. or white spot syndrome virus (WSSV). Non-lethal heat shock (NLHS) has also been shown to facilitate a tolerance or resistance to the pathogens through various molecular factors^3,4^. For instance, the Hsp70 transcript is increased in the hepatopancreas of Chinese shrimp *Fenneropenaeus chinensis* after WSSV infection^5^. Hsp70 and Hsp90 mRNAs are also up-regulated in the gills of black tiger shrimp *Penaeus monodon* upon *Vibrio harveyi* infection^6^. In *L. vannamei, Lv*Hsp60 and *Lv*Hsp70 proteins are significantly up-regulated and expressed in the gills, hepatopancreas, and hemocytes after bacterial challenge^7^. These findings from different shrimp species reveal the conserved functional role of Hsps in shrimp and also highlight some aspects of a potential resistance mechanism to pathogenic infection that is related to heat stress in shrimp, and perhaps in invertebrates in general.

MicroRNAs (miRNAs) are small non-coding RNA molecules that play an important role in RNA silencing and post-transcriptional regulation^8^. The action of miRNAs begin when a mature miRNA is incorporated into the RNA-induced silencing complex (RISC), resulting in specific interactions with target mRNAs. The complementary target mRNA is degraded and thus, translationally repressed^9^. Previous small RNA-Seq study has identified differentially expressed miRNAs from *L. vannamei* hemocytes upon VP_AHPND_ infection. 222 shrimp miRNA target genes (involved in various biological functions) that encode proteinase inhibitors, apoptosis-related proteins, and heat shock proteins were predicted^10^. Analysis of the expression of different miRNAs, in response to bacterial infections, indicates miRNAs are contributors in the host innate immune response, and thus help to illustrate the general role of miRNAs in immunity^11^.

In this study, the functional roles of miRNAs in immunity and stress survival are further explored by using RNA-Seq to investigate the global expression of mRNA and miRNA populations in the hemocytes of VP_AHPND_-infected shrimp that are pre-treated with NLHS. Several mRNAs and miRNAs were selected for expression analysis to verify the RNA-Seq data. The inferred relationships among the target genes and miRNAs will help to reveal important aspects of these molecules pertaining to AHPND resistance or tolerance, providing valuable insights into the modulation of immune pathways by NLHS.

## Results

### Effect of NLHS on shrimp survival upon VP_AHPND_ challenge

**Figure 1:**
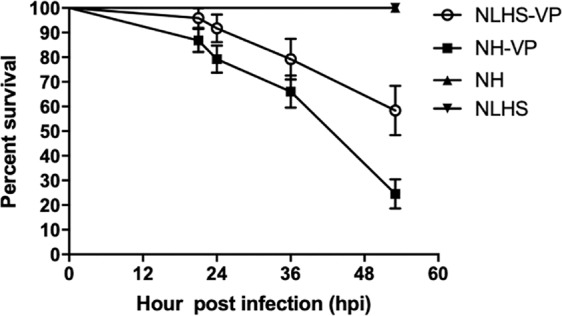
Effect of chronic non-lethal heat stress (NLHS) on the survival of VP_AHPND_-challenged shrimp. Shrimps were stressed under the NLHS condition or reared normally under the NH condition. After 3 days recovery, shrimp were infected with VP_AHNPD_ by immersion. The TSB contained 1.5% NaCl (instead) for the control group. Shrimp survival was measured every 6 h post-infection (hpi) for 53 h. NLHS-VP (○) and NH-VP (■) represent NLHS- and NH-treated shrimps challenged with VP_AHPND_, respectively. NLHS (▼) and NH (▲) represent NLHS- and NH-treated shrimp immersed with TSB containing 1.5% NaCl. The experiment was performed in triplicates, and the survival percentage was calculated as the mean ± 1 standard error (S.E.) at each time point.

Herein, we have confirmed that treating shrimp with NLHS prior to VP_AHPND_ infection results in a significantly higher survival rate, as previously demonstrated by Jungprung *et al*.^12^. This survival experiment was set-up by dividing shrimps into four groups of non-heat treatment control (NH), NLHS control (NLHS), VP_AHPND_ challenge (NH-VP), and NLHS plus VP_AHPND_ challenge (NLHS-VP) (Fig. 1). For the NLHS treatment group, shrimps were placed in tanks at 38 °C for 5 min daily for 7 days and allowed to recover in 30 °C tanks at ambient temperature, without any disturbance for 3 days. No mortality was observed until the end of the experiment in heat treatments without a VP_AHPND_ challenge. In those with VP_AHPND_ challenge, the survival rate was increased in the group with NLHS. In particular, the survival rate of the NH-VP group (24.53%) is significantly lower than the NLHS-VP (58.33%) group based on a Log-rank test, which indicates a possible heat-induced tolerance to bacterial infection in the NLHS-VP group.

### Gene expression profiling in *L. vannamei* hemocytes under the NLHS condition

The shrimp innate immune response upon NLHS treatment was analyzed in the hemocytes because it is the tissue in which the majority of immune reactions take place. Hemocytes from 30 individuals, each in the NH-VP and NLHS-VP groups, were collected at 0, 6, and 24 h post infection (hpi), pooled and used for cDNA library preparation. The experiments were completed as triplicates for a total of 18 cDNA libraries that were loaded into an Illumina Next-Seq 500 sequencer (Table S1). Raw sequence data from 18 normalized libraries were concatenated and analyzed. Average %Q30 and the sequence range were 81.975% and 30–151 bp, respectively. Total raw single pass reads for all the libraries amounted to 400,232,814 reads, which were reduced to 399,998,390 reads after additional adapter trimming, quality filtering and size selection (50–151 bp). Each library had an average number of filtered reads of 22,222,133. Sequencing reads were deposited in the Short Read Archive (SRA) of the National Centre for Biotechnology Information (NCBI) and can be accessed using accession numbers in Table S1.

**Figure 2:**
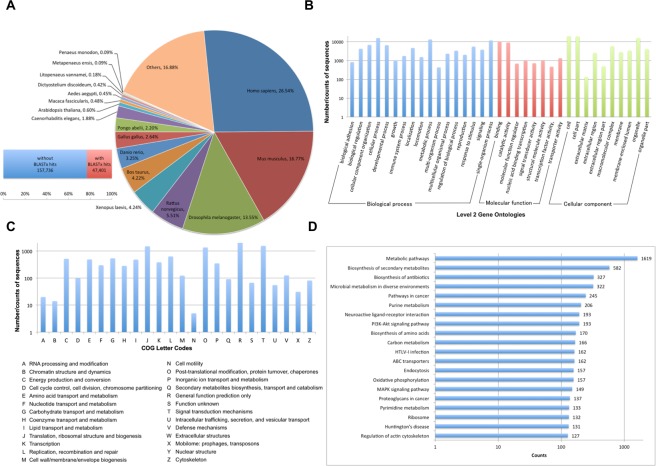
Sequence analysis and functional annotation of assembled unigenes identified from *L. vannamei* hemocytes under NLHS and NH conditions with and without VP_AHNPD_ challenge. (**A**) Number of BLAST hits (E-value < 10) in the reference transcriptome and sequence distribution in different organisms. (**B**) Number of sequences with hits according to level 2 gene ontologies (GO). (**C**) Number of sequences in the transcriptome assembly that are similar to the Cluster of orthologous groups (COGs). Letter codes with detailed descriptions are listed beneath the graph. (**D**) Top 20 KEGG pathways mapped in the assembly.

All clean reads from mRNA libraries were concatenated and *de novo* assembled using Trinity, which generated 174,835 putative genes (unigenes) or 205,137 isotigs. The generated reference assembly has an N50 isotig length of 1,074 represented by various isotigs ranging from 201 bp to 22,966 bp (Table S2). The isotigs/transcripts were annotated by searching their sequences using BLAST against transcripts predicted from the available *L. vannamei* genome in NCBI Genbank, Swiss-Prot, GO, Cluster of Orthologous Groups (COG), and KEGG Pathway databases. A total of 47,401 (or 23.11%) sequences had significant hits (E-value ≤ 10) to the Swiss-Prot database and the majority of these sequences were homologous to *Homo sapiens* (26.54%), *Mus musculus* (16.77%), and *Drosophila melanogaster* (13.55%) genes (Fig. 2A). BLAST2GO mapped 184,422 level 2 gene ontologies (Fig. 2B), while COGs classified 11,350 sequences into different categories (Fig. 2C). Searching against the KEGG database showed that 33,475 sequences were mapped to a KEGG orthology, but only 20,183 were grouped into the reference pathways. The metabolic pathways, biosynthesis of secondary metabolites and biosynthesis of antibiotics were among the top 20 KEGG pathways (Fig. 2D) represented in the transcriptome assembly. A protein BLAST was also completed using the predicted coding sequences from the Trinotate protocol and these annotations, along with other supplementary information such as the transmembrane regions are shown in Table S3.

### Differentially expressed genes (DEGs) in NLHS-treated *L. vannamei* upon VP_AHPND_ challenge

In this study, we aimed to study the effect of NLHS treatment on transcription in VP_AHPND_*-*challenged shrimp. Therefore, only NH-VP-responsive genes and NLHS-VP-responsive genes were identified. Differentially expressed genes (DEGs) in NLHS-treated *L. vannamei* (upon VP_AHPND_ challenge) were identified by pairwise comparisons among the relevant groups as shown in the volcano plots (Fig. S1). Three biological replicates were used in the experiments and differential gene expression is represented as fold change against a specific group (Table S4; Table S4_summary_fold-changes_mRNAs.xlsx). Between the VP_AHPND_ challenged groups at 0 hpi (0 NH-VP) and 6 hpi (6 NH-VP), 792 genes were differentially expressed and 272 of these genes were significantly up-regulated (FDR < 0.05) in 6 NH-VP, whereas 520 genes were down-regulated in 6 NH-VP. The gene, identified as *L. vannamei* Relish small isoform gene (FJ416145), had the highest up-regulation (362-fold) in this particular comparison. Between the 0 NH-VP and VP_AHPND_ challenged group at 24 hpi (24 NH-VP), 676 genes were differentially expressed; 224 and 452 genes were up- and down-regulated, respectively, in the 24 NH-VP group. The Relish small isoform gene was also up-regulated in this group (144-fold), together with *P. monodon* triosephosphate isomerase gene homolog (7.4-fold). In the NLHS-treated shrimp challenged with VP_AHPND_ at 0 hpi (0 NLHS-VP) vs 6 hpi (6 NLHS-VP) comparison, 522 genes were differentially expressed, and 262 of these were significantly up-regulated in the 6 NLHS-VP group, whereas 260 genes were down-regulated. The gene homolog of lipoprotein aminopeptidase was found to be 5.3-fold up-regulated in the 6 NLHS-VP group, 3.9-fold up-regulated in the 6 NH-VP group, and 3.7-fold up-regulated in the 24 NH-VP group. Between the 0 NLHS-VP and 24 NLHS-VP, 272 genes were up-regulated in the 24 NLHS-VP group, whereas 520 genes were down-regulated.

### Sequence analysis of shrimp miRNAs

To supplement the information derived from the transcriptome, we also explored the global miRNA expression to obtain a glimpse of some regulators that are associated with the observed gene expression. To do this, we analyzed the miRNAs expressed in VP_AHPND_**-**infected and NLHS-treated shrimp (mir_NLHS-VP) by sequencing them at various sampling times (0, 6, and 24 h post infection; hpi). High-throughput sequencing generated 1,086,629 total raw reads in the 0 mir_NLHS-VP, 879,272 in the 6 mir_NLHS-VP, and 1,114,328 in the 24 mir_NLHS-VP. The high-quality sequences that passed initial quality filters were 948,089, 771,799, and 956,249 reads, respectively.

**Figure 3:**
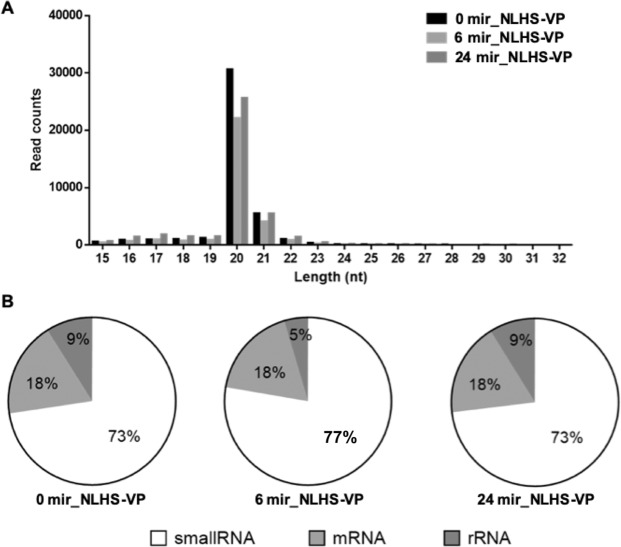
Length distribution, abundance and composition of small RNA libraries of VP_AHPND_-infected and NLHS-treated *L. vannamei* hemocytes. (**A**) Length distribution and abundance of small RNAs from hemocytes of NLHS-treated *L. vannamei* infected with VP_AHPND_ at 0 (0 mir-NLHS-VP), 6 (6 mir-NLHS-VP), and 24 (24 mir-NLHS-VP) hpi. (**B**) Composition of RNAs in each small RNA-Seq library.

Final filtering and analysis generated a majority of the non-redundant sequences 20–22 nucleotides (nt) in length (Fig. 3A). Searching the NCBI nucleotide database revealed that, on average, 25% of the sequences are most likely contaminating RNAs (Fig. 3B). After removal of these contaminating mRNA, rRNA, and tRNA homologs, the final counts of sequences were 78,000, which were mapped to miRBase 22.1 generating 77,415 sequences with hits. The percentages of matched mature miRNA sequences were 93.86%, 93.93%, and 94.19%, respectively, for the 0 mir_NLHS-VP, 6 mir_NLHS-VP, and 24 mir_NLHS-VP libraries. Sequences with unknown identities and homologs were also listed. Of those, forty-one miRNA homologs were identified from the NLHS-VP experimental group (Table S5).

### RT-qPCR validation of significant differentially expressed miRNAs (DEMs) and DEGs

In order to confirm the presence of the identified miRNAs and mRNAs, as well as to analyze the expression of interesting *L. vannamei* miRNAs and mRNAs in response to VP_AHPND_ infection under NLHS and control conditions, the expression profiles of 10 DEMs (lva-miR-7170-5p, lva-miR-2169-3p, lva-miR-184, lva-miR-92b-5p, lva-miR-317, lva-miR-92a-3p, lva-miR-4901, lva-miR-61, lva-miR-2898, and lva-miR-6090) and 8 DEGs (relish, lipoprotein receptor, dynamin, importin7, juvenile hormone epoxide hydroxylase 1; JHEH-1, DNAJ5, prophenoloxidase 1; PO1, and prophenoloxidase 2; PO2) that were identified from the sequencing data, were analyzed for their expression using RT-qPCR.

**Figure 4:**
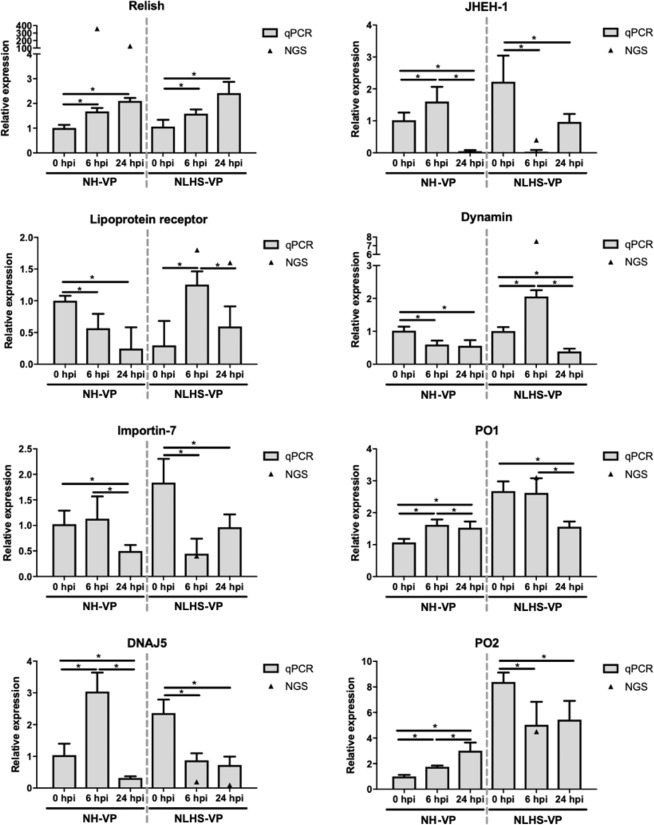
Validation of RNA-Seq using RT-qPCR. Eight representative genes (relish, lipoprotein receptor, dynamin, importin7, juvenile hormone epoxide hydroxylase 1; JHEH-1, DNAJ5, prophenoloxidase 1; PO1, and prophenoloxidase 2; PO2) were evaluated for their expression in hemocytes of shrimp under the NLHS and NH conditions in response to VP_AHPND_ infection and are referred to as NLHS-VP and NH-VP, respectively. Total RNA from hemocytes of NLHS-VP and NH-VP *L. vannamei* at 0, 6, and 24 hpi was used for cDNA synthesis. The relative expression levels of eight genes were determined by RT-qPCR and normalized against EF-1α, the internal reference. The relative expression ratio was calculated using the 2^−ΔΔCT^ method. The experiments were completed using triplicates. The expression level was calculated relative to that of the normal shrimp under the NH condition at 0 h after the VP_AHPND_ challenge. The bar graphs are the data from RT-qPCR presented as means ± standard deviations and the triangles (▲) are data from the RNA-Seq. Asterisks indicate significant difference (*P* < 0.05) from the respective VP_AHPND_ infected NH shrimp at 0 hpi.

Under the NLHS-VP condition, Relish gene expression was significantly higher in all experimental groups than the respective controls. The dynamin gene was up-regulated 2-fold at 6 hpi and down-regulated 2-fold at 24 hpi. The lipoprotein receptor gene was up-regulated nearly 2-fold at 6 hpi. Importin7, JHEH-1, DNAJ5, PO1, and PO2 were significantly down-regulated 1.5- to 10-fold at 6 hpi and 24 hpi (Fig. 4). It should be noted, the expression pattern determined from the RNA-Seq data was similar to the expression pattern of selected DEGs determined from the RT-qPCR results.

**Figure 5:**
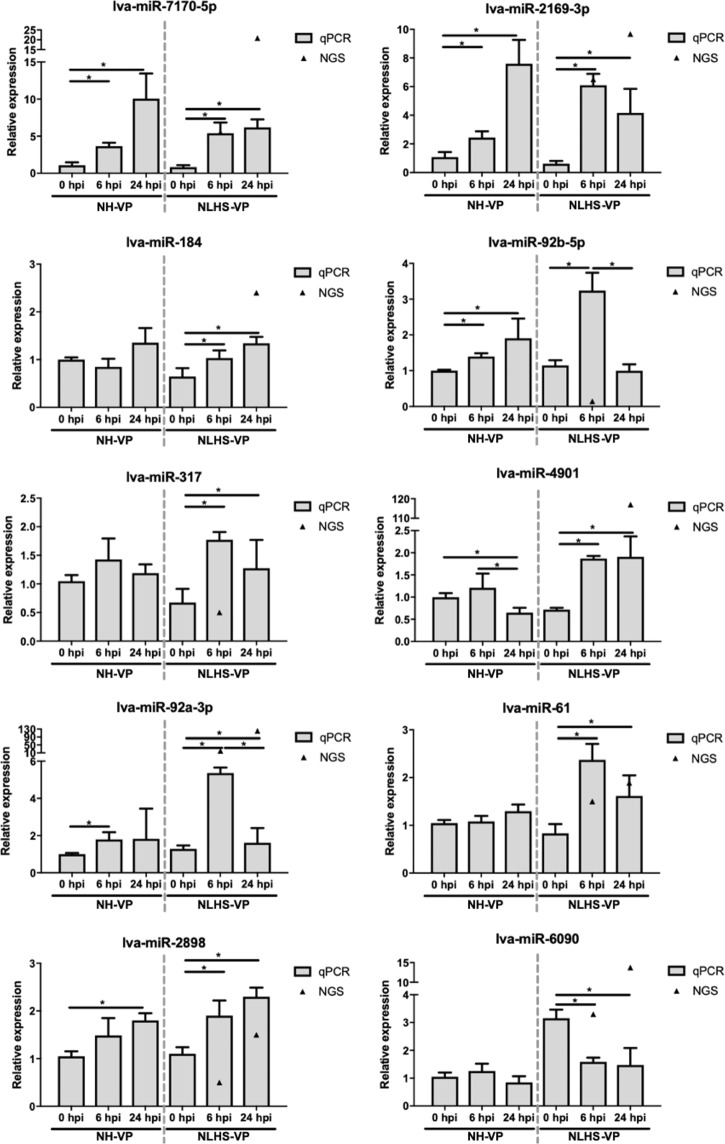
Relative expression analysis of miRNAs in response to VP_AHPND_ infection following the NLHS and NH treatments in *L. vannamei* hemocyte. Total small RNAs from hemocyte of VP_AHPND_-infected *L. vannamei* under NH- and NLHS-treated conditions which are NH-VP and NLHS-VP, respectively, were used as templates for specific stem-loop first strand cDNA synthesis. Relative expression levels of 10 miRNAs (lva-miR-7170-5p, lva-miR-2169-3p, lva-miR-184, lva-miR-92b-5p, lva-miR-317, lva-miR-4901, lva-miR-92a-3p, lva-miR-61, lva-miR-2898, and lva-miR-6090) were determined by RT-qPCR and normalized against U6, the internal reference, at 0, 6, and 24 hpi. The bar graphs are data from RT-qPCR presented as means ± standard deviations and triangles (▲) are data from the small RNA-Seq. The results were derived from triplicate experiments. Asterisks indicate significant differences (*P* < 0.05) from the respective VP_AHPND_-infected shrimp at 0 hpi.

Meanwhile, stem loop RT-qPCR analysis revealed that the expression levels of all 10 chosen DEMs were altered in shrimp hemocytes following NLHS treatment and VP_AHPND_ challenge by about 1.5- to 8-fold. For the NH-VP condition, only some of these DEMs had significant changes in their expression levels. These were lva-miR-2898, lva-miR-2169-3p, lva-miR-7170-5p, and lva-miR-92b-5p, which were all up-regulated at 6 and 24 hpi by around 1.5- to 10-fold, repectively (Fig. 5). The expression of 7 of 10 selected miRNAs were significantly altered under the NLHS-VP condition, also consistent with the small RNA-Seq data.

### Correlation of DEMs and DEGs of NLHS-treated shrimp in response to VP_AHPND_ infection

**Figure 6:**
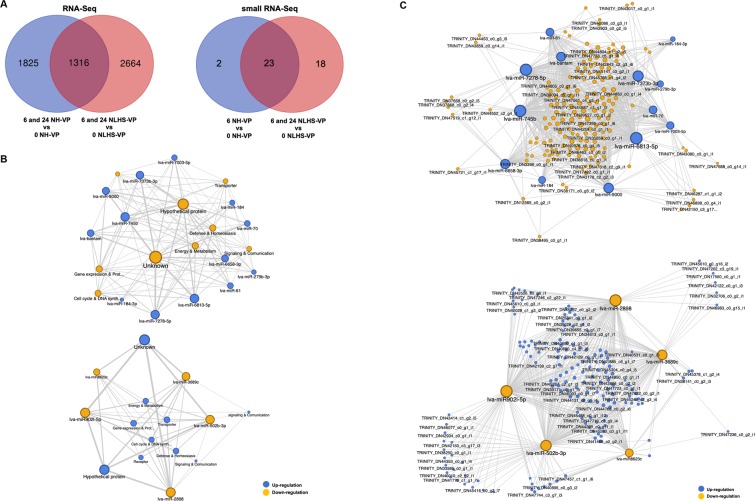
Network analysis for miRNA/mRNA interaction. (**A**) The Venn diagrams represent number of unique and common *L. vannamei* mRNAs and miRNAs that are expressed in response to VP_AHPND_ infection and following the NLHS and NH treatments. The differentially expressed genes (DEGs) and miRNAs (DEMs) were identified based on RNA-Seq data and small RNA-Seq data, respectively. The number of DEGs or DEMs identified through the normalization of genes or miRNAs expressed by *L. vannamei* hemocytes infected with VP_AHPND_ (NLHS-VP) at 6 and 24 hpi (6 NLHS-VP and 24 NLHS-VP) to that of 0 hpi (0 NLHS-VP) is shown in the red circle. The blue circle represents the number of DEGs or DEMs derived from VP_AHPND_-infected *L. vannamei* hemocyte controls (NH-VP) at 6 and 24 hpi (6 NH-VP and 24 NH-VP) with that of 0 hpi (0 NH-VP). (**B**) The miRNA/mRNA negative correlation network based on the predicted miRNA target function. The upper panel is the miRNA/mRNA network of up-regulated miRNAs (blue circle) and down-regulated genes (yellow circle), whereas the lower panel is the miRNA/mRNA network of down-regulated miRNAs (yellow circle) and up-regulated genes (blue circle). (**C**) The miRNA/mRNA negative correlation network of selected unique isotigs. The upper panel is the miRNA/mRNA network of up-regulated miRNAs (blue circle) and down-regulated genes (yellow circle), whereas the lower panel is the miRNA/mRNA network of down-regulated miRNAs (yellow circle) and up-regulated genes (blue circle). The circular nodes represent mRNAs and miRNAs. The degree of connectivity, which represents the number of genes regulated by a given miRNA, is indicated by the size of the node.

The DEGs of 3,980 NLHS-VP-responsive genes and 3,141 NH-VP-responsive genes were compared and used to construct a Venn diagram highlighting specific groups of DEGs (Fig. 6A). A grouping of 2,664 DEGs were considered to be the NLHS-VP-responsive genes, while another grouping of 1,825 DEGs were categorized as the NH-VP-responsive genes. Their intersection with 1,316 DEGs were expressed in response to VP_AHPND_ infection whether or not the shrimp were treated with NLHS. In the future, it will be interesting to analyze the NLHS- and VP-responsive genes to broaden our knowledge regarding the shrimp immune response against NLHS and VP_AHPND_ infection.

The DEMs of NLHS-VP analyzed in this study and of NH-VP identified in our previous work^13^ were analyzed to further identify miRNAs that regulate immune genes of NLHS-treated shrimp infected with VP_AHPND_. As with the expression profiles of the DEMs, a Venn diagram was also created to highlight specific groupings of DEMs between the libraries of NLHS-VP and NH-VP. Eighteen DEMs were specifically grouped into NLHS-VP-responsive miRNAs and two DEMs were added to a NH-VP-responsive miRNAs group (Fig. 6A).

The DEMs from the NLHS-VP-responsive miRNAs that group only with their corresponding target mRNAs from the sequencing dataset were analyzed using CU-mir (in-house) and RNA-hybrid software. This analysis facilitated the identification and functionalizing of specific miRNA-mRNA interactions, which then served as a clue to the general regulatory mechanisms underlying the immune response of shrimp under the NLHS and VP_AHPND_ infection. 1,833 DEM-DEG pairs with negative correlations were identified and included in a miRNA-target network (Table S6). Some of the biological functions that might be regulated by the NLHS-VP miRNAs were found to include: “Defense & Homeostasis”, “Energy & Metabolism”, “Cell cycle & DNA Synthesis/repair”, “Gene expression & Protein synthesis/degradation”, “Receptor”, “Signaling & Communication”, “Transporter”, “Hypothetical protein”, and “Unknown”. Several miRNAs such as lva-miR-7278-5p, lva-miR-6813-5p, lva-miR-745b, lva-miR902l-5p, lva-miR-502b-3p, and lva-miR-2898 had high degrees of connectivity and might play crucial roles in the regulatory network. Meanwhile, genes involved in “Defense & Homeostasis”, “Energy & Metabolism”, and “Cell cycle & DNA Synthesis/repair” were the most common miRNA targets (Fig. 6B,C).

In order to further characterize the identified biological pathways, an enrichment analysis specific for immune-related pathways was done for the identified DEGs of miRNA-mRNA pairs. Three pathways that changed significantly (*P*-value < 0.05) were “hemocyte homeostasis”, “prophenoloxidase system”, and “AMP production”. Given these immune pathways and the information on the canonical members of these pathways (in related studies), we were able to find sequence homologs of these members in our RNA-Seq dataset. We then used publicly available information on shrimp as well as other sequences in our transcriptome data to further annotate these homologs. This sequence information was used to lookup gene expression patterns in the identified pathways using RT-qPCR.

**Figure 7:**
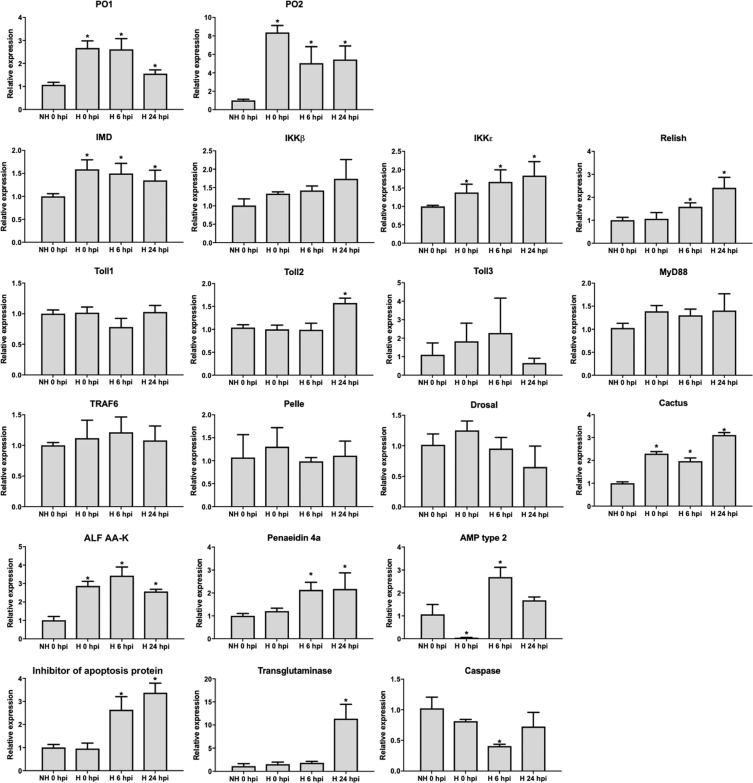
Relative expression analysis of genes in the NLHS-VP modulated immune pathways. Expression level of genes in the prophenoloxidase system (PO1 and PO2), IMD pathway (IMD, IKKε, IKKβ, and Relish), Toll pathway (Toll1, Toll2, Toll3, MyD88, TRAF6, Pelle, Drosal, and Cactus), antimicrobial peptide (ALF AA-K, Penaedin 4a, and AMP type 2), and hemocyte homeostasis (TGase and Inhibitor of apoptosis protein) was determined by RT-qPCR in the hemocytes of VP_AHPND-_challenged *L. vannamei* at 0 hpi (NH 0 hpi) and of NLHS-treated *L. vannamei* challenged with VP_AHPND_ at 0 hpi (H 0 hpi), 6 hpi (H 6 hpi), and 24 hpi (H 24 hpi). Relative expression ratios are calculated using EF-1α as the internal control. Relative expression level of each gene in hemocytes of NLHS-treated *L. vannamei* challenged with VP_AHPND_ at each time point after infection was normalized to that of NH 0 hpi to determine the effect of both NLHS and VP_AHPND_ challenge. The results were derived from triplicate experiments. Asterisks indicate significant differences at *P* < 0.05 from the NH 0 hpi. The expression profile of PO1 and PO2 under the NLHS condition was modified and re-presented from Fig. 4.

**Figure 8:**
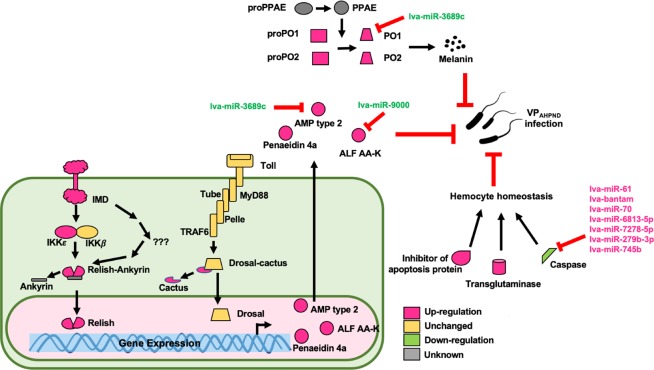
A schematic representation of how the NLHS-VP-responsive miRNAs modulate the shrimp immune responses upon NLHS treatment and VP_AHPND_ infection. This is based on the predicted interactions between miRNAs and target immune genes of *L. vannamei* under the NLHS-treated and VP_AHPND_-challenged condition.

Figure 7 shows the expression profiles of some canonical members of pathways identified in this study. It is of note that Fig. 7 presents the expression profile of PO1 and PO2 under the NLHS condition, which is also presented in Fig. 4, which reveals that PO1 and PO2 of the prophenoloxidase system are highly up-regulated, from 1.5- to 8-fold, in the NLHS-VP group. Looking at the members of the IMD pathway, IMD, IKKβ, and Relish, all had higher gene expression in the NLHS-VP group. Meanwhile, Toll1, Toll2, Toll3, MyD88, TRAF6, Pelle, and Dorsal genes in the Toll pathway were not significantly changed in the NLHS-VP group. For the hemocyte homeostasis pathway, transglutaminase and inhibitor of apoptosis protein were analyzed and found to be highly up-regulated while the caspase gene was down-regulated in the NLHS-VP group. Confirming the results of the pathway enrichment analysis, these findings suggest that the biological immune pathways, “hemocyte homeostasis”, “prophenoloxidase system”, and “AMP production via IMD pathway”, might play important roles in the enhancement of shrimp antibacterial immunity against VP_AHPND_ upon modulation of NLHS (Fig. 8). The NLHS trigger could be considered a prior conditioning and preparation mechanism for the shrimp to fight later infections, thereby enhancing bacterial immunity against VP_AHPND_ and improving survival rates.

## Discussion

The AHPND is known to be caused by VP_AHPND_, which accumulates in the stomach of shrimp and secretes PirA/B toxins in the hepatopancreas^14^. The mechanism by which AHPND kills shrimp is currently unclear. Recent data demonstrates that the genes of Toll and IMD pathways, and their downstream antimicrobial peptides (AMPs) are suppressed in the stomach and in hemocytes, but are overexpressed in the hepatopancreas^15^. This implies that while the stomach and hepatopancreas are major VP_AHPND_ targets, the hemocytes, being the major immune organs of shrimp, may also provide informative clues regarding the immune mechanisms against disease^16^.

The previous observation that the non-lethal heat shock enhances the production of heat shock proteins and, subsequently, increases the expression of some immune-related genes resulting in enhanced immunity^4^, is of research interest because of its potential application in developing preventive strategies for diseases in shrimp. For instance, a short-term hyperthermic treatment that is suggested to reduce gill-associated virus replication in *P. monodon*, may prove to be a simple and effective prophylactic strategy^17^.

In this study, we found that NLHS improved the survival of VP_AHPND_-infected *L. vannamei* (Fig. 1), revealing that the NLHS may indeed modulate immune factors to alleviate the mortality caused by AHPND. However, previous studies have shown that the tolerance and survival rate of *L. vannamei* after VP_AHPND_ infection is not influenced by Hsp70 accumulation or changes in immune-related proteins, such as proPO and hemocyanin^4^. This indicates that the mechanisms of resistance to VP_AHPND_ infection under the NLHS conditions remain unexplored and may (potentially) be explained by some undescribed immune proteins. Thus, we performed RNA-Seq and small RNA-Seq analyses of either NLHS-treated or NH control shrimp infected by VP_AHPND_ to explore the genes, gene networks, and miRNAs that regulate these unknown immune mechanisms.

Here, sequencing of mRNAs generated approximately 400 million (400 M) reads across 18 libraries for *de novo* assembly, which is 4-times and 6-times higher than those of recent transcriptome libraries from the hepatopancreas^18^ and stomach^19^, respectively. These 18 libraries represent 3 biological replicates with an average of 22 M reads, each with 6 samples ensuring optimum statistical power to detect differentially expressed genes^20^. We also assembled a higher number of 205,137 isotigs with higher 1,074 N_50_ isotig length. Regarding the number of unigene sequences with COG and KEGG annotations, this study includes a higher number of hits compared to Rao, *et al*.^18^ However, the percentage of BLASTx hits of our reference assembly to the SWISS-Prot database and the *L. vannamei* genome-transcribed data was lower (23.11% vs 26.13%)^18^. The percentage (26.89%) of unknown contigs in our assembly may indicate that we mined more novel genes than previously described, a collection of interesting genes related to immune response and bacterial tolerance to characterize in the future.

Small RNA libraries prepared from the NLHS-treated and VP_AHPND_-infected *L. vannamei* hemocytes (NLHS-VP) generated 3 million reads across 3 libraries, which revealed 41 DEM homologs, and 27 up-regulated and 14 down-regulated miRNAs. Previously, there were 47 up-regulated and 36 down-regulated miRNA homologs identified in VP_AHPND_ challenged *L. vannamei* hemocytes^10^. In this study, eight miRNA homologs that included lva-miR-184, lva-let-7, lva-miR-9, lva-miR-305, lva-miR-71, lva-miR-2, lva-miR-274, and lva-miR-317 were found to be DEMs, similar to a previous study from 2018^10^. Predicted target genes of NLHS-VP-responsive miRNA homologs such as caspase, c-type lectin, and Kazal-type serine proteinase inhibitor were similar to those of VP_AHPND_-responsive miRNAs identified in a previous study^10^, confirming the roles of NLHS-VP-responsive miRNAs in the regulation of immune-related genes in shrimp.

The RNA-Seq data was useful in identifying genes associated with bacterial infection and NLHS response and in providing sequence data, which can be used to predict the interactions with miRNAs. Relish, a gene identified in the sequencing dataset as a NH-VP-responsive gene, was found to be highly expressed in both NH-VP and NLHS-VP groups based on RT-qPCR confirmation. This indicates that the RNA-seq dataset can correctly identify candidate genes that are of significant relevance to the experimental treatment. Nevertheless, it should be noted that the observed profiles from the RNA-seq dataset could only be detected during either heat stress or bacterial infection (but not both), as demonstrated by RT-qPCR. For example, the dynamin and lipoprotein receptor were predicted in the RNA-Seq dataset to be up-regulated genes in the NLHS-VP group. The RT-qPCR expression analysis confirmed this, but also demonstrated a similar expression profile in the NH-VP group. Likewise, the JHEH-1, importin-7, and DNAJ5 were predicted to be down-regulated genes in the NLHS-VP dataset and were again confirmed by RT-qPCR. However, these genes also showed the same down-regulated expression in the NH-VP group. The RNA-Seq data, therefore, needs validation by RT-qPCR to confirm the mechanisms of immune modulation that are specific for NLHS and for bacterial infection.

Gene expression data from the RNA-seq and RT-qPCR analyses also identified some genes that are up-regulated after bacterial infection when there is a prior NLHS applied to shrimp. A synergistic effect could be seen in the expression of some of these genes, e.g. lipoprotein receptor and dynamin, whose expression were not significantly changed in NH-VP, but then changed in the NLHS-VP group. This finding also supports that there is an immune modulation mechanism by NLHS prior to infection.

The RT-qPCR analysis of the expression of 10 selected miRNAs validated their predicted expression profiles in the RNA-Seq dataset (Fig. 5). Through the use of datasets containing DEMs and DEGs as well as miRNA-targeting information, we acquired different miRNA-mRNA pairs. Negative correlations were identified in the miRNA-mRNA pairs, which can be considered as evidence of miRNA targeting^21^. In a previous study, a total of 407 miRNA-mRNA interaction sites were predicted from the VP_AHPND_-infected *L. vannamei*. Among these, 11 DEMs with regulatory roles on 37 DEGs related to immunological responses to VP_AHPND_ infection were predicted^10^. This previous target prediction of VP_AHPND_-responsive miRNAs showed that the VP_AHPND_-responsive miRNAs might regulate caspase in the apoptosis pathway, trypsin, Kazal-type serine proteinase inhibitor, c-type lectin, chitinase, and lectin 3^10^. In this study, 18 DEMs were identified as unique NLHS-VP-responsive miRNAs, including lva-miR-61, lva-miR-3689c, lva-miR-6658-3p, lva-miR902l-5p, lva-miR8623c, lva-miR-184, lva-miR-184-3p, lva-miR-7373b-3p, lva-miR-502b-3p, lva-miR-9000, lva-miR-6813-5p, lva-miR-2898, lva-miR-70, lva-miR-279b-3p, lva-miR-7278-5p, lva-miR-7003-5p, lva-miR-745b, and lva-bantam. Among the interactions identified, 11 unique NLHS-VP-responsive miRNAs were predicted to regulate shrimp immune pathways that include hemocyte homeostasis, the prophenoloxidase system, and AMP production, which is proposed in Fig. 8. Currently, this and previous studies^10^ have identified novel and unique miRNA homologs in shrimp that are involved in the regulation of the immune system.

Within the interactome, the NLHS-VP-responsive miRNAs were mapped against DEGs. In this study, lva-miR-6813-5p, lva-miR-7278-5p, and lva-miR-745b were found to be down-regulated miRNAs that target the highest number of up-regulated NLHS-VP-responsive genes. Meanwhile, lva-miR-2898 and lva-miR-902l-5p were found to be up-regulated miRNAs that target the highest number of the down-regulated NLHS-VP-responsive genes.

As previously demonstrated, several miRNAs can target the same genes. Interestingly, several up-regulated miRNAs such as lva-miR-61, lva-bantam, lva-miR-70, lva-miR-6813-5p, lva-miR-7278-5p, lva-miR-279b-3p, and lva-miR-745b were predicted to target caspase, which is a protease enzyme that plays essential roles in programmed cell death for most crustacean species^22–24^. The down-regulation of caspase at 6 hpi, regulated by miRNAs upon NLHS and VP_AHPND_ infection, suggests that a lower number hemocyte might undergo apoptosis.

In hemocyte homeostasis, caspase and transglutaminase (TGase) are two important proteins for most crustacean species^22,23^. TGase transcript expression in NLHS-treated shrimp was up-regulated at 24 hpi upon VP_AHPND_ challenge. This expression profile is expected because TGase is known to be involved in hemolymph coagulation^25^. Experimental data showed that suppression of TGase results in low hemocyte counts and high bacterial counts in *M. japonicas*^25^, which supports that TGase is an important immune factor during bacterial infection. Up-regulation of TGase prior to bacterial infection is a good indication of how NLHS modulates the shrimp immune system in order to better survive the infection. At the same time, the down-regulation of caspase and up-regulation of inhibitor of apoptosis transcripts might synergize with the regulation of TGase by suppressing the apoptosis pathway. This suggests that NLHS-treated shrimp can better maintain hemocyte homeostasis during VP_AHNPD_ infection.

Phenoloxidase (PO) is a key enzyme in the proPO-system that triggers the non-enzymatic conversion of phenolic substances to quinones, leading to the production of cytotoxic intermediates and melanin^26^. Also known as the melanization cascade, the prophenoloxidase system is a major innate defense system in invertebrates that involves the melanization of pathogens and damaged tissues^27^. When the system is suppressed by gene silencing in shrimp, it increases susceptibility to bacterial infection^28^. The prophenoloxidase system was identified in this study as putatively regulated by the NLHS-VP-responsive miRNAs. This pathway was examined through the expression of its canonical members, such as PO1, a putative lva-miR-3689c target. Analysis by RT-qPCR showed that NLHS treatment causes down-regulation of lva-miR-3689c and up-regulation of PO1 at 24 h post VP_AHPND_ infection, suggesting that the proPO system is modulated by a lva-miR-3689c/PO1 interaction. This demonstrates that the lva-miR-3689c/PO1 interaction plays a crucial role in bacterial defense that leads to some form of resistance against the VP_AHPND_ infection. Up-regulation of the two *Lv*PO transcripts in *L. vannamei* (identified in a previous study) indicates an increase in disease resistance to VP_AHPND_^29^, which is consistent with the results presented here.

The last identified pathway putatively regulated by the NLHS-VP is the antimicrobial peptide/protein (AMP) production. Shrimp AMPs are diverse and generally cationic peptides that primarily protect the host against a broad range of microorganisms. Because they also have a secondary role in modulating other immune effectors and different biological pathways, their synthesis and induction is governed by a complex interplay of various factors. Specifically, the Toll and IMD pathways are regarded as key regulating mechanisms in the transcription of AMP genes^21^. The anti-lipopolysaccharide factor (ALF) is one of major shrimp AMPs and its expression is regulated by the Toll and IMD pathways^30,31^. In this study, the transcription of ALF was found to be modulated by NLHS, along with other AMPs such as penaeidin and AMP type 2. These results consistently support AMP’s importance in the immune mechanisms governing VP_AHPND_ infection and VP_AHPND_ toxin resistance^32,33^. According to the miRNA/mRNA network, lva-miR-3869c and lva-miR-9000 are predicted regulators of AMP type 2 and ALF AA-K, respectively, indicating the crucial roles of miRNA in modulating AMP gene expression in NLHS-treated shrimp prior to VP_AHPND_ infection. To further characterize the regulatory mechanisms of the Toll and IMD pathways, the expression of the genes related to these pathways were analyzed. Analysis of the IMD pathway showed that transcripts of IMD, IKKε and Relish are highly expressed in the NLHS-VP shrimp, consistent with the expression of downstream genes, such as penaeidin. Previous studies have found that the high expression of IMD after *V. anguillarum* infection is an important regulatory mechanism in shrimp immunity against Gram-negative bacteria^34^. The IMD expression observed in the current study is consistent with the hypothesis of immune modulation by NLHS.

In conclusion, an integrated analysis of miRNAs and mRNAs can effectively elucidate the mechanisms that govern the tolerance of shrimp to VP_AHPND_ infection after NLHS treatment. The interaction of NLHS-VP miRNAs to their predicted mRNA targets may have a strong regulatory influence on the 3 immune-related pathways in shrimp, although these interactions will need to be directly tested. By providing new insights regarding the regulatory roles of miRNAs in the biological changes that occur in shrimp during NLHS and bacterial infection, the present study improves our understanding of the mechanisms that underlie NLHS treatment and mediate the immunity of shrimp against VP_AHPND_ infection. Once we understand how NLHS activate the shrimp immune pathway to fight VP_AHPND_ infection, biomolecules that can activate those immune pathways can be further identified and applied in the field.

## Materials and Methods

### Ethics statement

According to the Ethical Principles and Guidelines for the use of animals for scientific purposes by the National Research Council of Thailand, the experiments involving animals were carried out and complied with animal use protocol number 1823006 approved by the Chulalongkorn University Animal Care and Use Committee (CU-ACUC).

The biosecurity concerns of this study were reviewed and approved by the Institutional Biosafety Committee & Chulalongkorn University (CU-IBC) (Approval number: SCI-01-001) and are in accordance with the levels of risk in pathogens and animal toxins listed in “the Risk Group of Pathogen and Animal Toxin (2017)” issued by the Department of Medical Sciences, Ministry of Public Health, the Pathogen and Animal Toxin Act (2015) and Biosafety Guidelines for Modern Biotechnology, BIOTEC (2016).

### Shrimp samples

Healthy shrimps weighing 2–4 grams were obtained from a local shrimp farm and acclimatized in rearing tanks with an ambient temperature of 30 °C, water salinity of 20 parts per thousand (ppt) and constant aeration before use in experiments. The shrimps were fed with commercial pellets 4 times a day during the course of the experiments. The shrimps were sampled to determine if they were free from VP_AHPND_ and WSSV-infections by PCR prior to experiments using the specific primer as described by Sirikharin *et al.*^35^ and Tummamunkong *et al.*^36^ (Fig. S2).

### Non-lethal heat stress (NLHS) and bacterial challenge experiments

The VP_AHPND_ inoculum was prepared by culturing bacteria overnight in 3 mL of tryptic soy broth (TSB) containing 1.5% NaCl at 30 °C and 250 rpm. Then, the starter culture was transferred to 200 mL TSB with 1.5% NaCl and further incubated at 30 °C and 250 rpm until the OD_600_ reached 2.0 (approximately 10^8^ CFU/mL).

After rearing at 30 °C, the chronic non-lethal heat stress (NLHS) was applied to the shrimp. The shrimps were divided into four groups of 10 shrimps each. Two groups were NLHS-treated by placing the shrimp in tanks containing 10 L of sea water at 38 °C for 5 min daily for 7 days, shrimp were given a 3-day recovery period in their respective rearing tanks. The other two groups were reared in the tank at the ambient temperature (30 °C) as control groups of non-heat (NH) treatment. Shrimp were then challenged with VP_AHPND_ by immersion in tanks containing the bacterial ioculum at a final concentration of 1.5 × 10^6^ CFU/mL. The uninfected control group was immersed in the TSB containing 1.5% NaCl. The shrimp survival was observed for 53 h. Experiment was completed in triplicates. Statistical analyses of the results were conducted using GraphPad Prism version 6. The infographic outlining all experimental groups is shown in Fig. S3.

### RNA extraction

NLHS and bacterial challenge experiments were performed as described above and approximately 500 µL of hemolymph of VP_AHPND_-challenged NLHS-treated and VP_AHPND_-challenged NH control shrimp at 0, 6, and 24 h post infection (hpi) time points were drawn out from the ventral sinus using a sterile syringe pre-loaded with an equal volume of anticoagulant (27 mM sodium citrate, 336 mM NaCl, 115 mM glucose, and 9 mM EDTA, pH 5.6)^37^. Hemocytes were immediately collected by centrifugation at 800 × *g* for 10 min at 4 °C and kept in liquid nitrogen. The hemocytes from 30 individuals were pooled and extracted for large and small RNAs using the mirVana miRNA Isolation Kit (Ambion, Life Technologies) following the manufacturer’s protocol. These experiments were done using triplicates. RNA integrity was evaluated using the Agilent 2100 Bioanalyzer chip, RNA 6000 Pico Kit and Small RNA Kit (Agilent) for large and small RNA preparations, respectively. The RNA concentrations were determined using the Qubit RNA HS Assay Kit on the Qubit 2.0 flourometer (ThermoFisher Scientific).

### RNA sequencing (RNA-Seq) and data analysis

Six cDNA libraries that included 0 NLHS-VP, 6 NLHS-VP, 24 NLHS-VP, 0 NH-VP, 6 NH-VP, and 24 NH-VP with three biological replicates were prepared from 4 μg total RNA following the manufacturer’s protocol for TruSeq Stranded mRNA LT Sample Prep Kit (Illumina). Eighteen indexed libraries were normalized, pooled and then sequenced with a 1% PhiX spike-in control using the NextSeq 500 Mid Output v2 Sequencing Kit (Illumina) in a NextSeq 500 desktop sequencer (Illumina). Additional adapter trimming and quality control of raw reads was performed using the FastQ Toolkit available through the BaseSpace (Illumina) public app repository. High quality reads were assembled to form a reference assembly in Trinity v2.06 software^38^. Transcript abundance was estimated using RSEM wrapped by scripts included in Trinity. Differentially expressed genes (DEGs) were detected using the edgeR software^39^ and R program^40^ for each treatment group and checked for sequence quality and correlation (Fig. S4). The DEGs were selected based on FDR < 0.05 and fold change >2. Pairwise comparisons between relevant groups were analyzed using 3 biological replicates and expressed as the fold change against a specific group. Bootstraps and permutation resampling were set to a default (500) as well as the largest genes size (5000). No correction was applied to FDR, but only FDRs lower than 0.05 were considered significant and listed in Table S4. The *de novo* assembled sequences to the *L. vannamei* transcripts predicted from *L. vannamei* genome data available in GenBank (https://www.ncbi.nlm.nih.gov/genome/?term = vannamei).

Gene ontology enrichment analysis for differentially expressed features was done using the Trinotate protocol (http://trinotate.github.io/), leveraging different scripts and software for functional annotation, such as BLASTx^41^, PFAM^42^, HMMER^43^, SignalP^44^, tmHMM^45^, KEGG Orthology^46^, GO^47^, and eggNOG^48^, and, then, running GO-Seq^49^. Using the UniProt Retrieve/ID mapping tool (http://www.uniprot.org/uploadlists/), the UniProt accession numbers from the Trinotate protocol were mapped into the Entrez GeneIDs, which were then used in KOBAS 2.0^50^ (http://kobas.cbi.pku.edu.cn/index.php) to map the KEGG Orthology or conduct the enrichment analysis. The subsequent KEGG Orthology was then used as input in the KEGG Mapper – Search Pathway tool (http://www.kegg.jp/kegg/tool/map_pathway1.html) for mapping to the reference KEGG pathways and determining the distribution^51–53^. The BLAST2GO^54^ was also used for some supplementary annotation. The Fasta tools, Trinity software^38,55^, BLAST + ^41,56,57^ and other supplementary tools from the Galaxy services of the National Center for Genome Analysis Support (https://galaxy.ncgas-trinity.indiana.edu/)^58,59^ and the Galaxy Queensland (https://usegalaxy.org.au/)^60^ were also used.

### Small RNA-Seq and data analysis

The cDNA libraries from small RNA from VP_AHPND_-infected NLHS-treated shrimp hemocytes at 0, 6, and 24 hpi were constructed following the manufacturer’s instruction and the TruSeq Small RNA Library Preparation Kit (Illumina). Three indexed libraries were normalized, pooled, and sequenced with a PhiX control spiked at 7.5% using MiSeq Reagent Kits v2 (Illumina) in a MiSeq sequencer (Illumina). The 5′-, 3′-adapter trimming and quality control of raw reads were performed using tools in a Galaxy instance (https://usegalaxy.org/)^58^. High quality small RNA sequences with lengths shorter than 18 nucleotides, and longer than 24 nucleotides, were removed. Homology search for contaminating RNA, such as mRNA, rRNA, and tRNA was conducted using BLASTn against the NCBI nucleotide and Rfam database. After discarding the contaminating RNA, the remaining sequences were searched against miRBase 22.1 (http://www.mirbase.org/) in order to identify known miRNA homologs. Based on the number of reads from 3 libraries cut off >10, the miRNA homologs were selected for the differentially expressed miRNA (DEM) analysis. The specific procedures were as follows: (1) treatment and control groups were normalized to the same orders of magnitude. Formula: Normalized expression level = miRNA expression level/total expression level of the sample × normalized magnitude; (2) Normalized results were used to calculate the fold change and *P*-value^61^.

### Quantitative real-time PCR analysis

Several transcripts from the reference assembly were selected for quantitative real-time PCR analysis (RT-qPCR) to evaluate and confirm the differential expression profiles reported by RNA-seq analysis. The gene specific primers (Table S7) were designed by Primer3 as packaged in Geneious R6 (Biomatters)^62^. Using 1 µg total RNA, the first strand cDNA synthesis was carried out in a reaction containing 1 mM dNTP, 10 units of RNase inhibitor, 0.5 µM oligo-dT (Promega), 1 × RevertUP buffer (BiotechRabbit), and 100 units of RevertUP Reverse Transcriptase (BiotechRabbit). The RT-qPCR reactions comprised 5- or 10-fold diluted cDNA template, 1 × QPCR Green Master Mix (LRox) (BiotechRabbit) and 0.5 or 0.25 µM forward and reverse primers, and were run in a MiniOpticon Real-time PCR system (Bio-Rad). The relative expression of each gene was determined and analyzed using EF-1α gene as an internal control gene. The expression levels from the zero-time-point of non-heat-treated shrimps upon VP_AHPND_ infection (0 NH-VP) were used as a control group.

The miRNAs of interest consisting of lva-miR-7170-5p, lva-miR-2169-3p, lva-miR-184, lva-miR-92b-5p, lva-miR-317, lva-miR-92a-3p, lva-miR-4901, lva-miR-61, lva-miR-2898, and lva-miR-6090 were selected for expression analysis using stem-loop RT-qPCR. The pooled total small RNA samples from the VP_AHPND_-infected NLHS-treated (NLHS-VP) and control shrimp hemocytes (NH-VP) at 0, 6, and 24 hpi were prepared using the mirVana miRNA Isolation Kit (ThermoFisher Scientific). The extracted total small RNA was then used as a template for the first strand stem-loop cDNA synthesis using the stem-loop primers (Table S7) by RevertAid First Strand cDNA Synthesis Kit (ThermoFisher Scientific). The U6 gene expression was used as an internal control^63^. Stem-loop RT-qPCR was performed using the RT-qPCR reactions comprised of 2- or 5-fold diluted cDNA templates for each miRNA specific oligonucleotide primers (Table S7), and QPCR Green Master Mix (BiotechRabbit) in the MiniOpticon RealTime PCR System (Bio-Rad) under the following conditions: 95 °C for 3 min, 40 cycles of 95 °C for 30 s, 60 °C for 30 s, and 72 °C for 30 s. Relative expression was calculated and data were analyzed using paired-sample t-tests and are presented as means ± standard deviations^64^. The statistical significance was determined if *P*-values were <0.05. Experiment were performed in triplicates.

### miRNA target prediction

The miRNA targets were identified by comparing the miRNA sequences with transcriptome data using CU-Mir software developed by our research group (http://shrimp-irn.org/mirtarget/index.php)^63^. The software searched for the sequences on mRNA that match (perfectly) or mismatch (by one nucleotide) the seed sequences (2–8 nucleotides from the 5′-end) of miRNA. The percent complementary was calculated from the number of nucleotides that perfectly match the target mRNAs per total length of miRNA sequences. The percent total length complementary cutoff was set at 55%. The RNAhybrid (http://bibiserv.techfak.uni-bielefeld.de/rnahybrid/) was also used to predict genes targeted by the miRNAs with the parameters of free energy <−15.0 kcal/mol^65^.

### miRNA/mRNA interaction network analysis

In order to define all possible miRNA-mRNA interactions involved in a specific dataset of immune-related genes, only NLHS-VP-responsive DEGs and DEMs with negative correlations were grouped. NLHS-VP-responsive DEMs were used as queries to search for mRNA targets from NLHS-VP-responsive DEGs. Again, the miRNA/mRNA binding were predicted using RNAhybrid with the parameters of free energy <−15 kcal/mol. These target mRNAs were mapped against RNA-Seq data to determine their gene functions. Subsequently, the miRNAs/mRNA pairs involved in a specific dataset of immune-related genes were included in the integrated analysis of the NLHS-VP miRNA/mRNA network. The workflow of data integration approach used to build the shrimp NLHS-VP miRNA/mRNA network is shown in Fig. S5.

## Supplementary information

Supplementary information.

Supplementary information 2.

Supplementary information 3.

Supplementary information 4.

Supplementary information 5.

Supplementary information 6.
