# Supplementary material for: MicroRNA and mRNA interactions coordinate the immune response in non-lethal heat stressed Litopenaeus vannamei against AHPND-causing Vibrio parahaemolyticus
Source: Sci Rep. 2020 Jan 21;10:787. doi: 10.1038/s41598-019-57409-4 (PMC6972907; doi:10.1038/s41598-019-57409-4)
Supplement: Supplementary file 1 — Supplementary information. [file 41598_2019_57409_MOESM1_ESM.pdf]

MicroRNA and mRNA interactions coordinate the immune response in non-lethal heat stressed *Litopenaeus vannamei* against AHPND-causing *Vibrio parahaemolyticus*

Pakpoom Boonchuen[1], Benedict A. Maralit[1,A], Phattarunda Jaree[1,3], Anchalee Tassanakajon[1,2], Kunlaya Somboonwiwat[1,2,*]

[1]Center of Excellence for Molecular Biology and Genomics of Shrimp, Department of Biochemistry, Faculty of Science, Chulalongkorn University, Thailand

[2]Omics Science and Bioinformatics Center, Faculty of Science, Chulalongkorn University, Thailand

[3]Institute of Molecular Biosciences, Mahidol University, Salaya, Nakhon Pathom Thailand

[A]Current Addresses: Philippine Genome Center, University of the Philippines, Diliman, Quezon City, Philippines; and National Institute of Molecular Biology and Biotechnology, College of Science, University of the Philippines, Diliman, Quezon City, Philippines

* Corresponding author: kunlaya.s@chula.ac.th

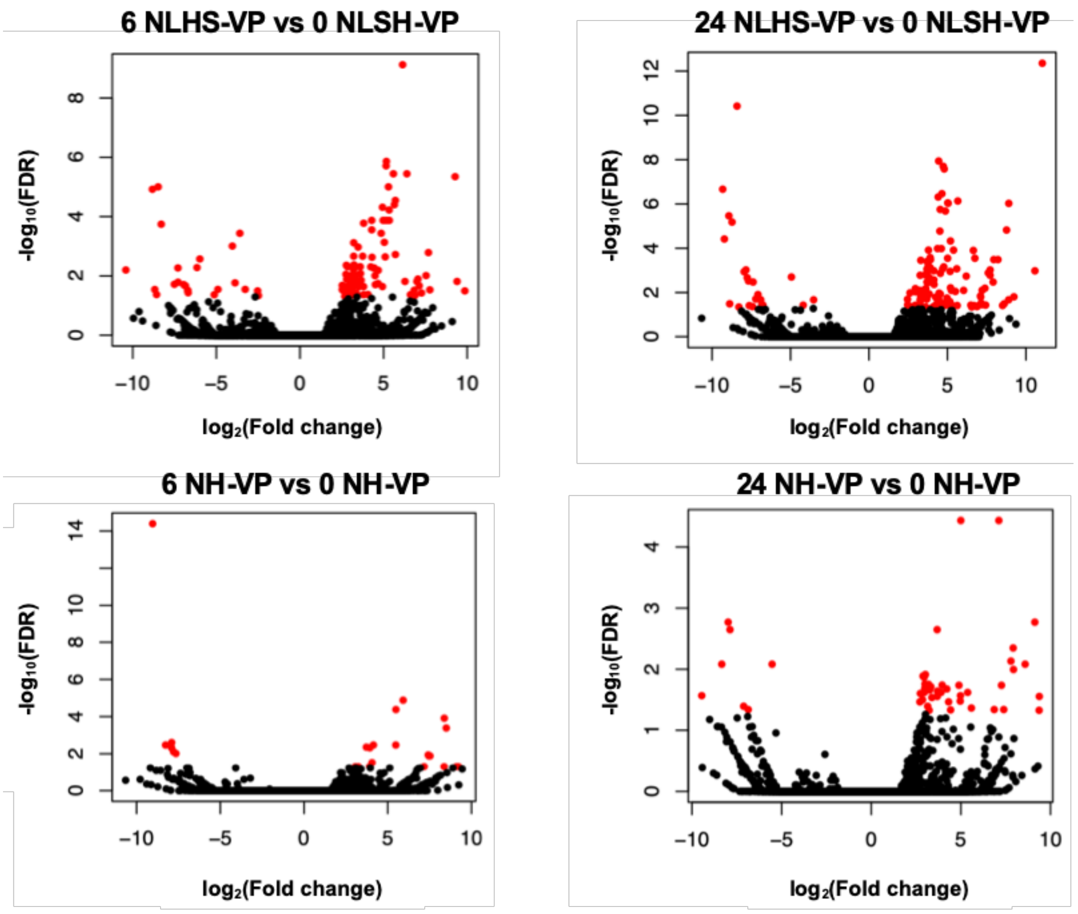

Fig. S1 Volcano plots illustrating $\log_{10}$(FDR) in relation to the $\log_2$(Fold change) for 6 NLHS-VP and 24 NLHS-VP conditions versus 0 NLHS-VP control condition and 6 NH-VP and 24 NH-VP conditions versus 0 NH-VP control condition. Genes that passed the significance threshold FDR adjusted *P*-value < 0.05 and the expression cut-off $\log_2$(fold change) > 2 are colored red, while genes outside this range are colored black.

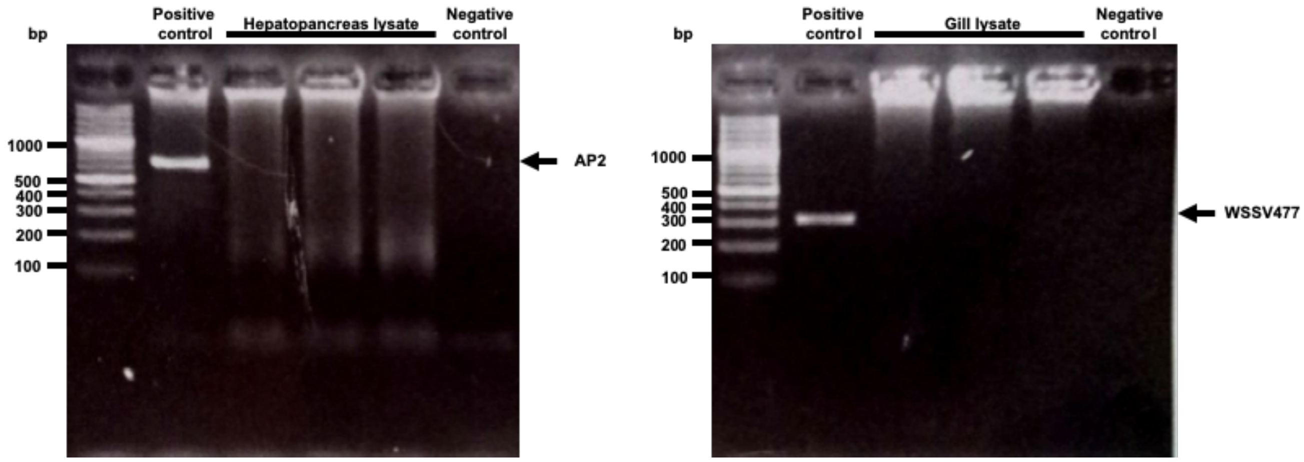

Fig. S2 Verification of non-VP_{AHPND} and -WSSV-infected shrimp samples by PCR. Gills and hepatopancreas of 3 shrimps from the local shrimp farm were collected and ground in the sterile water. One µl of either gill and hepatopancreas lysate was used as template for WSSV and VP_{AHPND} detections, respectively, by PCR using the specific primers (AP2 for VP_{AHPND} and WSSV477 for WSSV).

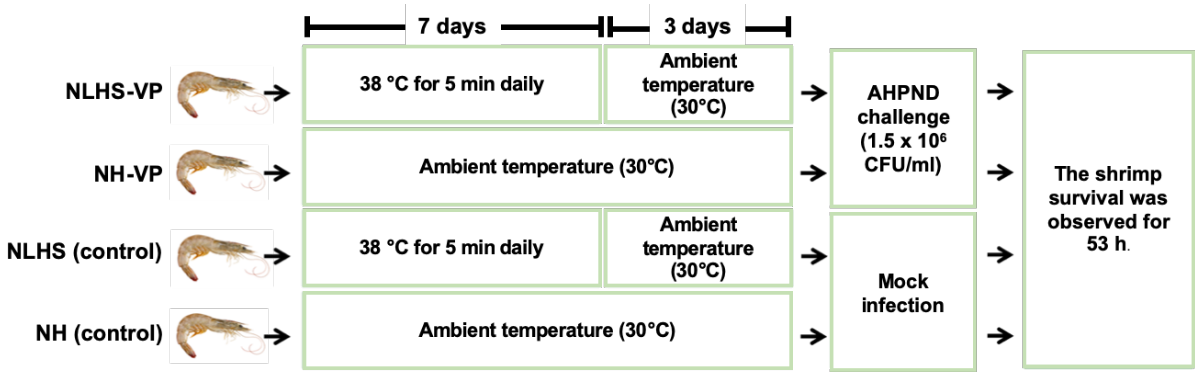

Fig. S3 The shrimp were divided into four groups of 10 shrimp each. Two groups were NLHS-treated by placing shrimps in tanks containing 10 L sea water at 38 °C for 5 min daily for 7 days and allowed a 3-day recovery period in their respective rearing tanks. The orther two groups were reared in the tank at 30 °C as control groups of non-heat treatment. They were, then, challenged with VP_{AHPND} by immersion in VP_{AHPND} inculum at a final concentration of $1.5\ 10^6$ CFU/mL. TSB containing 1.5% NaCl was used instead of VP_{AHPND} inoculum in the control. The shrimp survival was observed for 53 h. The experiment was done in triplicate.

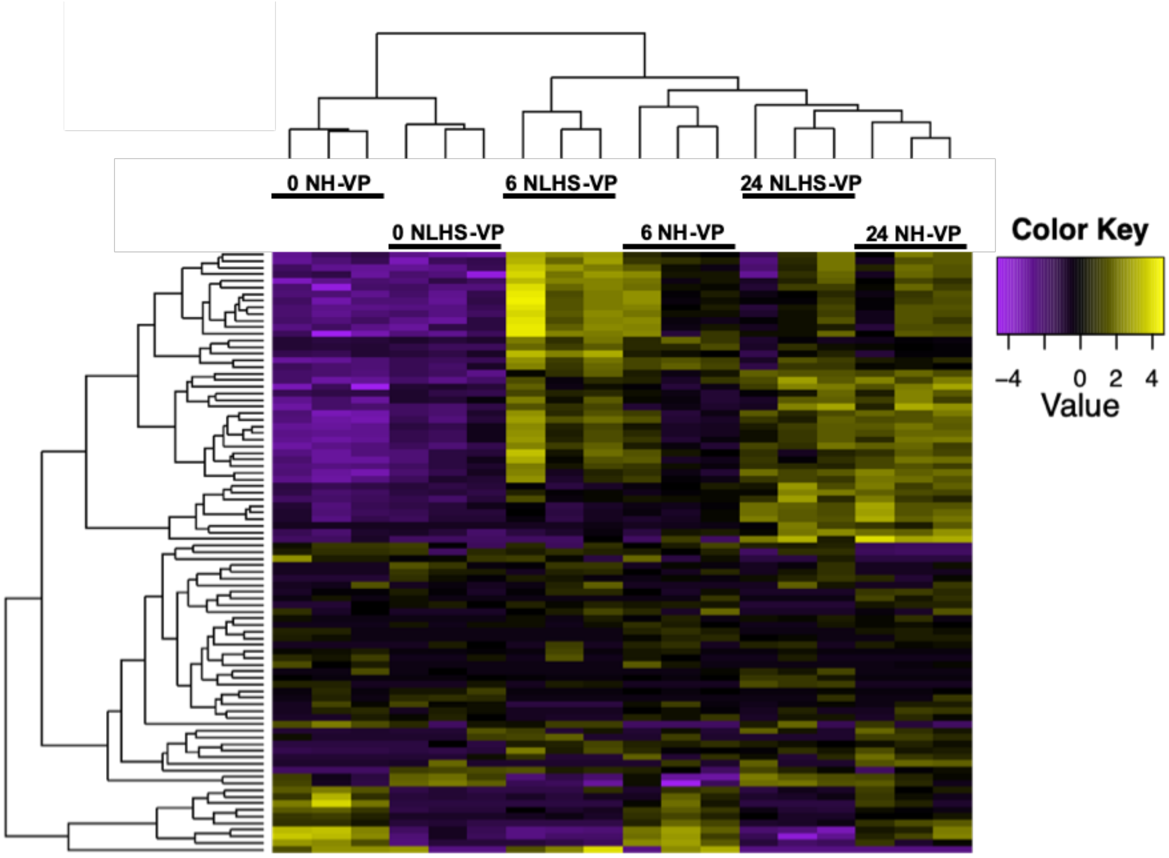

Fig. S4 Hierarchy-clustered heatmap of (TMM-normalized) FPKM expression values for those genes (represented in rows) that are at least 2-fold differentially expressed (*P*-value <0.001).

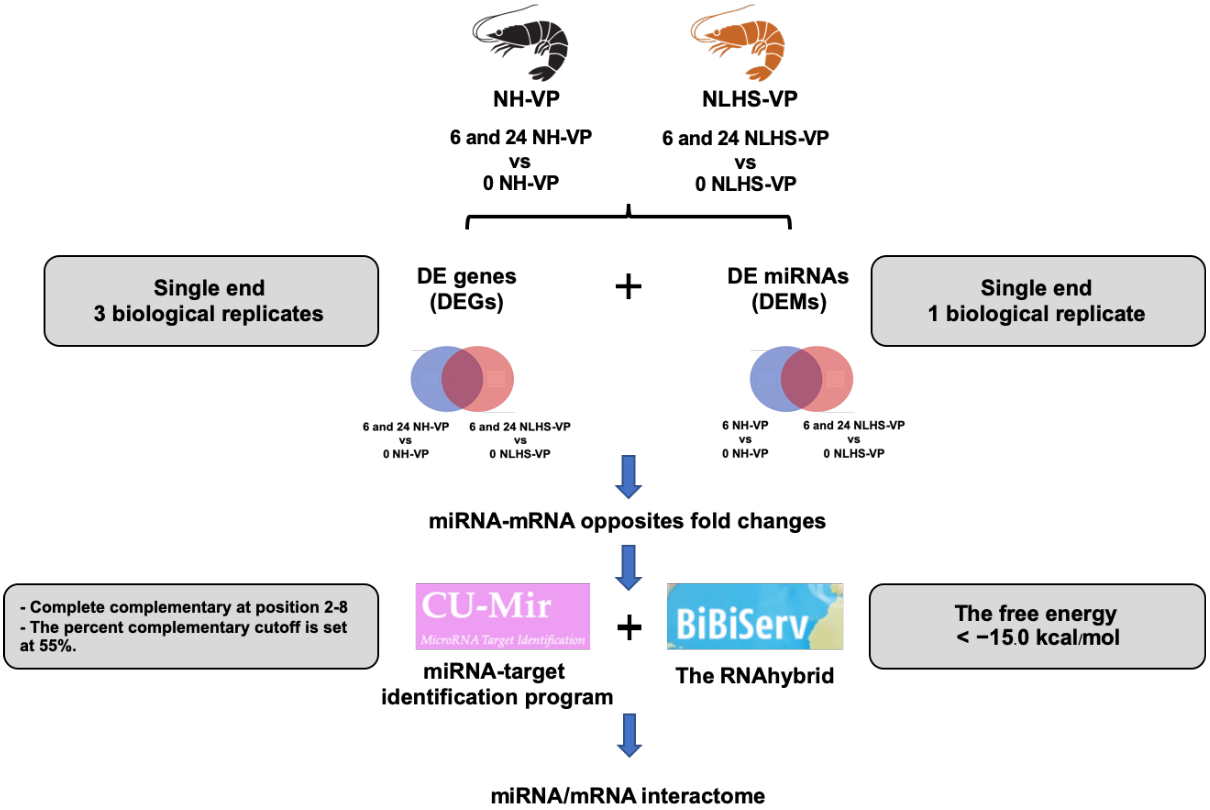

Fig. S5 Data integration approach. The differentially expressed miRNAs (DEMs) and genes (DEGs) with negative correlation were correlated and integrated. Further, miRNA-mRNA interactions from prediction tools were integrated. Finally, target genes that followed these criteria were considered to build the shrimp NLHS-VP miRNA/mRNA network.

Table S1 Number of sequences obtained from RNA-Seq in each cDNA library of *L. vannamei* hemocytes after NH or NLHS treatment and VP_{AHNPD} infection.

ID	Condition	Time Point (hpi)	Replicate No.	Number of reads	Accession numbers
**0NH-VP**	non-heat	0	replicate 1	24,914,546	SRR3993813
**0NH-VP**	non-heat	0	replicate 2	18,790,695	SRR3997616
**0NH-VP**	non-heat	0	replicate 3	20,783,002	SRR3997617
**6NH-VP**	non-heat	6	replicate 1	22,850,301	SRR3997618
**6NH-VP**	non-heat	6	replicate 2	23,616,635	SRR3997619
**6NH-VP**	non-heat	6	replicate 3	22,809,007	SRR3997620
**24NH-VP**	non-heat	24	replicate 1	23,665,724	SRR3997621
**24NH-VP**	non-heat	24	replicate 2	23,782,913	SRR3997622
**24NH-VP**	non-heat	24	replicate 3	23,155,408	SRR3997623
**0 NLHS-VP**	heat	0	replicate 1	23,480,445	SRR3997631
**0 NLHS-VP**	heat	0	replicate 2	20,214,421	SRR3998867
**0 NLHS-VP**	heat	0	replicate 3	21,731,765	SRR3998869
**6 NLHS-VP**	heat	6	replicate 1	24,793,063	SRR3998870
**6 NLHS-VP**	heat	6	replicate 2	21,750,763	SRR3998871
**6 NLHS-VP**	heat	6	replicate 3	20,503,721	SRR3998872
**24 NLHS-VP**	heat	24	replicate 1	21,347,015	SRR3998873
**24 NLHS-VP**	heat	24	replicate 2	20,746,438	SRR3998874
**24 NLHS-VP**	heat	24	replicate 3	21,296,952	SRR3998992
**Total**				400,232,814	

Table S2 Trinity assembly statistics of RNA-Seq of *L. vannamei* hemocytes after NH or NLHS treatment and VP_{AHPND} infection.

Statistics for isotig lengths:	Value
Min isotig length:	201
Max isotig length:	22,966
Mean isotig length:	672.14
Median isotig length:	357
N50 isotig length:	1,074
**Statistics for numbers of isotigs:**	
Total trinity 'genes':	174,835
Number of isotigs:	205,137
Number of isotigs >=1kb:	31,106
Number of isotigs in N50:	28,762
**Statistics for bases in the isotigs:**	
Number of bases in all isotigs:	137,880,518
Number of bases in isotigs >=1kb:	71,369,224
GC Content of isotigs:	41.32%
